# Supplementary material for: Causal Relationship between Aging and Anorexia Nervosa: A White-Matter-Microstructure-Mediated Mendelian Randomization Analysis
Source: Biomedicines. 2024 Aug 16;12(8):1874. doi: 10.3390/biomedicines12081874 (PMC11351342; doi:10.3390/biomedicines12081874)
Supplement: Supplementary file 1 [file biomedicines-12-01874-s001.zip › Supplementary Figure/Figure S4/Singe SNP plot of MR analysis for TL to ubm-b-1944.pdf]

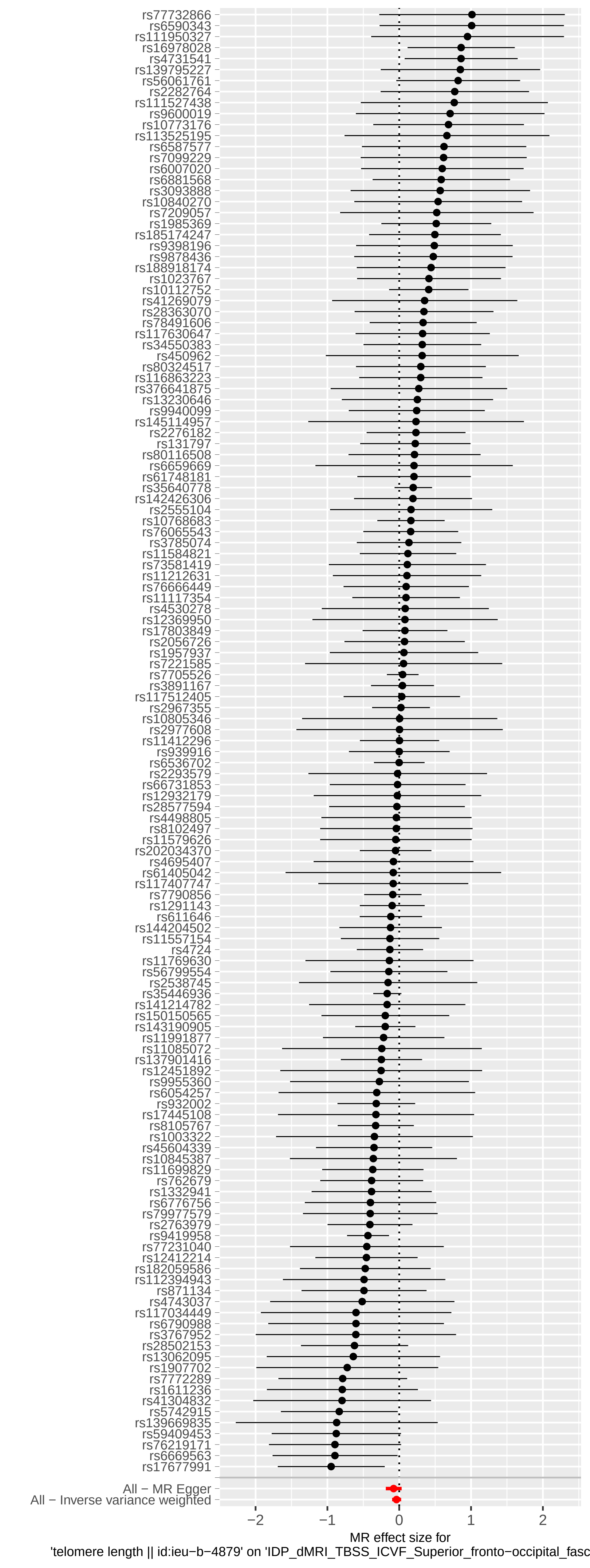
rs77732866
rs6590343
rs111950327
rs16978028
rs4731541
rs139795227
rs56061761
rs2282764
rs111527438
rs9600019
rs10773176
rs113525195
rs6587577
rs7099229
rs6007020
rs6881568
rs3093888
rs10840270
rs7209057
rs1985369
rs185174247
rs9398196
rs9878436
rs188918174
rs1023767
rs10112752
rs41269079
rs28363070
rs78491606
rs117630647
rs34550383
rs450962
rs80324517
rs116863223
rs376641875
rs13230646
rs9940099
rs145114957
rs2276182
rs131797
rs80116508
rs6659669
rs61748181
rs35640778
rs142426306
rs2555104
rs10768683
rs76065543
rs3785074
rs11584821
rs73581419
rs11212631
rs76666449
rs11117354
rs4530278
rs12369950
rs17803849
rs2056726
rs1957937
rs7221585
rs7705526
rs3891167
rs117512405
rs2967355
rs10805346
rs2977608
rs11412296
rs939916
rs6536702
rs2293579
rs66731853
rs12932179
rs28577594
rs4498805
rs8102497
rs11579626
rs202034370
rs4695407
rs61405042
rs117407747
rs7790856
rs1291143
rs611646
rs144204502
rs11557154
rs4724
rs11769630
rs56799554
rs2538745
rs35446936
rs141214782
rs150150565
rs143190905
rs11991877
rs11085072
rs137901416
rs12451892
rs9955360
rs6054257
rs932002
rs17445108
rs8105767
rs1003322
rs45604339
rs10845387
rs11699829
rs762679
rs1332941
rs6776756
rs79977579
rs2763979
rs9419958
rs77231040
rs12412214
rs182059586
rs112394943
rs871134
rs4743037
rs117034449
rs6790988
rs3767952
rs28502153
rs13062095
rs1907702
rs7772289
rs1611236
rs41304832
rs5742915
rs139669835
rs59409453
rs76219171
rs6669563
rs17677991
All − MR Egger
All − Inverse variance weighted
−2
−1
0
1
2
MR effect size for
'telomere length || id:ieu−b−4879' on 'IDP_dMRI_TBSS_ICVF_Superior_fronto−occipital_fasc
